# Immediate Genetic and Epigenetic Changes in F1 Hybrids Parented by Species with Divergent Genomes in the Rice Genus (*Oryza*)

**DOI:** 10.1371/journal.pone.0132911

**Published:** 2015-07-24

**Authors:** Ying Wu, Yue Sun, Kun Shen, Shuai Sun, Jie Wang, Tingting Jiang, Shuai Cao, Samuel Manthi Josiah, Jinsong Pang, Xiuyun Lin, Bao Liu

**Affiliations:** 1 Key Laboratory of Molecular Epigenetics of MOE and Institute of Genetics and Cytology, Northeast Normal University, Changchun, China; 2 Jilin Academy of Agricultural Sciences, Changchun, China; Ben-Gurion University, ISRAEL

## Abstract

**Background:**

Inter-specific hybridization occurs frequently in higher plants, and represents a driving force of evolution and speciation. Inter-specific hybridization often induces genetic and epigenetic instabilities in the resultant homoploid hybrids or allopolyploids, a phenomenon known as genome shock. Although genetic and epigenetic consequences of hybridizations between rice subspecies (e.g., japonica and indica) and closely related species sharing the same AA genome have been extensively investigated, those of inter-specific hybridizations between more remote species with different genomes in the rice genus, Oryza, remain largely unknown.

**Methodology/Principal Findings:**

We investigated the immediate chromosomal and molecular genetic/epigenetic instability of three triploid F1 hybrids produced by inter-specific crossing between species with divergent genomes of *Oryza* by genomic in situ hybridization (GISH) and molecular marker analysis. Transcriptional and transpositional activity of several transposable elements (TEs) and methylation stability of their flanking regions were also assessed. We made the following principle findings: (*i*) all three triploid hybrids are stable in both chromosome number and gross structure; (*ii*) stochastic changes in both DNA sequence and methylation occurred in individual plants of all three triploid hybrids, but in general methylation changes occurred at lower frequencies than genetic changes; (*iii*) alteration in DNA methylation occurred to a greater extent in genomic loci flanking potentially active TEs than in randomly sampled loci; (*iv*) transcriptional activation of several TEs commonly occurred in all three hybrids but transpositional events were detected in a genetic context-dependent manner.

**Conclusions/Significance:**

Artificially constructed inter-specific hybrids of remotely related species with divergent genomes in genus *Oryza* are chromosomally stable but show immediate and highly stochastic genetic and epigenetic instabilities at the molecular level. These novel hybrids might provide a rich resource of genetic and epigenetic diversities for potential utilization in rice genetic improvements.

## Introduction

Interspecific hybridization and its attendant whole genome doubling (allopolyploidization) are important evolutionary players in higher plants [1–4]. Recent genetic and genomic studies have revealed a range of rapid genetic and epigenetic changes associated with nascent hybridization and polyploidization in many plant taxa including *Arabidopsis* [5, 6], wheat [7–11], *Brassica* [12, 13], *Spartina* [14, 15], and others [16, 17]. These findings have lent strong supports to the “genome shock” theory proposed by McClintock nearly 30 years ago [18]. These rapid genomic changes are diverse in scope, including nucleotide base changes, activation of transposable elements (TEs), and cytosine methylation alterations, which are often causally linked to changes in gene expression, function and new phenotypes [19–22].

The *Oryza* genus comprises two cultivated and 22 wild species, with 10 genome constitutions, i.e., AA, BB, CC, BBCC, CCDD, EE, FF, GG, HHJJ and HHKK [23]. In addition, two additional tetraploid species, *O*. *punctata* (genome BBCC) and *O*. *officinalis* (genome CCC’C’) have been identified (http://www.knowledgebank.irri.org/images/docs/wild-rice-taxonomy.pdf), rendering this genus containing four tetraploid species. The wild species of *Oryza* contain a rich reservoir of genetic variations that becomes increasingly important to sustain rice improvement for higher yield, increased resistance to biotic and abiotic stresses, and more efficient agro-chemical utilization.

Over the years, various intra- and inter-specific F1 hybrids have been successfully produced in *Oryza*, but so far most efforts are devoted to common wild rice (*O*. *rufipogon*) or other species with the same AA genome as cultivated Asian rice (*O*. *sativa*) [24, 25]. Recent studies have shown that even hybridizations between the two rice subspecies, *japonica* and *indica*, may induce extensive alterations in DNA methylation and gene expression as a result of interactions between the differentiated subspecies’ genomes [26–28] and the changes were further exaggerated after genome doubling [28]. According to the “genome shock” theory [18], the combination of more divergent genomes should produce a greater degree of genetic and epigenetic instabilities. Indeed, dramatic changes in DNA methylation and gene expression were detected in F1 hybrids between cultivated rice and wild rice *O*. *officinalis* (genome CC) [29], and in a set of recombinant inbred lines (RILs) derived from introgressive hybridization of rice and *Zizania* which belongs to a different genus from rice in the *Oryzea* tribe [30–33]. Recently, we found immediate and strong transcriptome shock occurred in an interspecific F1 triploid hybrid in *Oryza* [34]. To further explore the extent and generality of genetic and epigenetic instabilities that may result from hybridizations between more remotely related species in the *Oryza* genus, we conducted the present study. We used the same interspecific F1 triploid hybrid reported earlier [34] as well as two newly produced triploid hybrids by crossing the standard laboratory *japonica* rice cultivar Nipponbare as the maternal parent and each of two different wild tetraploid rice species containing different genomes as paternal parents, and assayed their chromosomal and molecular genetic/epigenetic instabilities. We document immediate but largely stochastic genetic and epigenetic changes in all three triploid F1 hybrids without entailing meiotic process.

## Results

### Production and phenotypic characterization of three sets of triploid F1 hybrids with different genome compositions in *Oryza*

**Fig 1:**
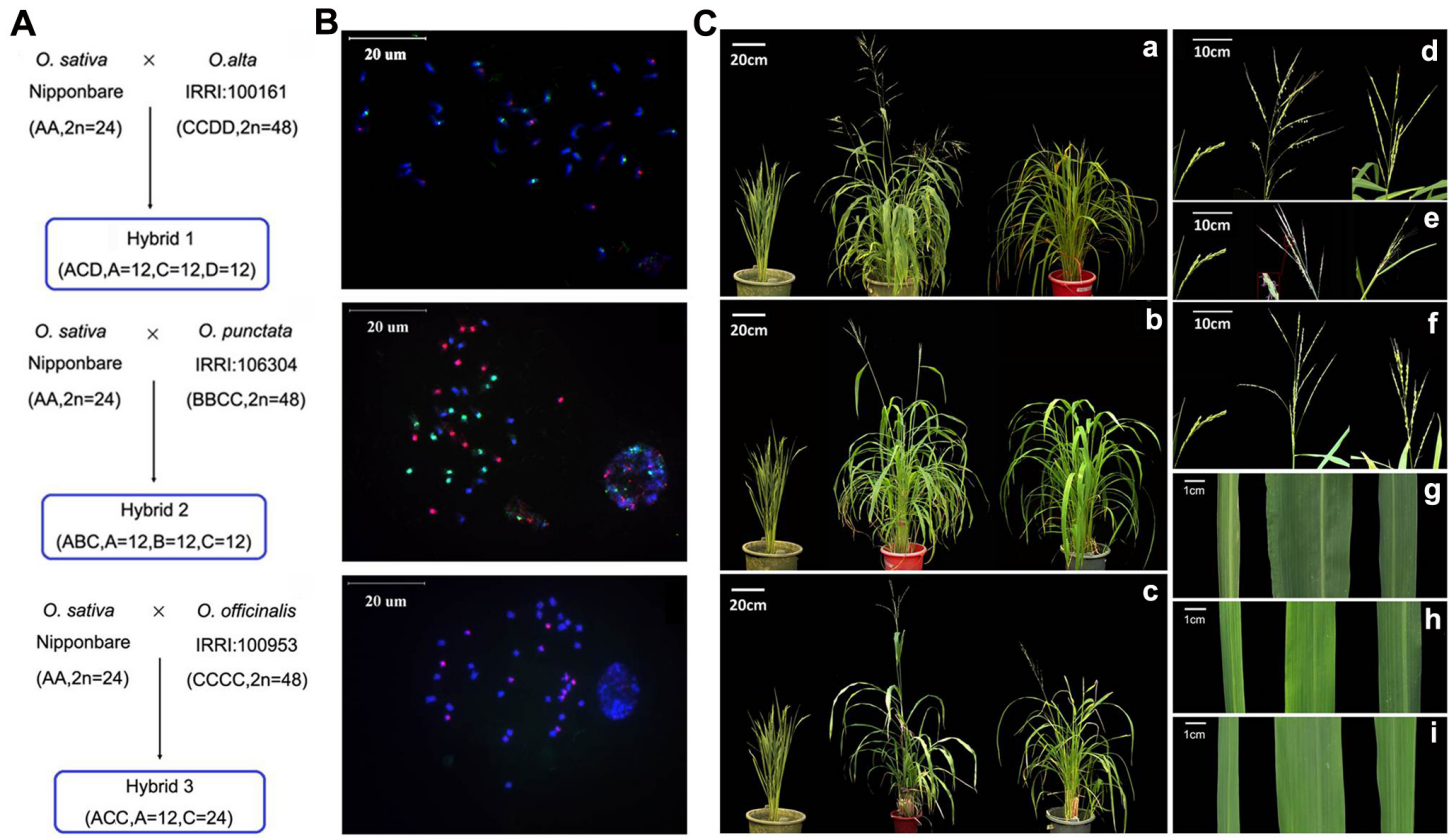
Schematic diagrams for three F1 rice hybrids, their genome compositions and several representative phenotypic traits. (A) Diagrams showing the parents and process for the construction of the three F1 hybrids, designated as Hybrid 1, Hybrid 2, and Hybrid 3, respectively. (B) Typical chromosomal constitutions of each of the three F1 hybrids, as revealed by multicolor GISH. The pink-, green-, and blue colored chromosomes represent the AA, CC, and the other genomes, respectively. The sphere-shaped clump is another intact nucleus in which though the chromosomes were not separated, their three coloration can also been seen. The photos from top to bottom are Hybrid1, Hybrid 2 and Hybrid 3, respectively. (C) Phenotypic characteristics of F1 hybrids and their parents; a, d, g are Hybrid 1, b, e, h are Hybrid 2, c, f, i are Hybrid 3. From left to right in Fig 1C were maternal Nipponbare, paternal wild rice and the corresponding F1 hybrids.

Three sets of triploid hybrids all with *Oryza sativa* ssp. *japonica*, cv. Nipponbare (genome AA, *2n* = 2x = 24) as the maternal parent and three different wild tetraploid rice species as the paternal parents were produced. Specifically, (1) crossing Nipponbare with *O*. *alta* (genome CCDD, *2n* = 4x = 48) to produce Hybrid 1 (genome ACD, *2n* = 3x = 36); (2) crossing Nipponbare with *O*. *punctata* (genome BBCC, *2n* = 4x = 48), to produce Hybrid 2 (genome ABC, *2n* = 3x = 36); and (3) crossing Nipponbare with *O*. *officinalis* (genome CCC’C’, *2n* = 4x = 48), to produce Hybrid 3 (genome ACC’, *2n* = 3x = 36). Hybrid plants of all three crosses were obtained by embryo rescue (**Fig 1A**). Hybrid nature of these plants was validated by genomic *in situ* hybridization (GISH) and chromosome counting (**Fig 1B**). No change in either chromosome number or gross structure was detected, indicating the hybrids were karyotypically stable. The hybrids were completely male sterile and showed overall intermediate plant statue between the parental species. However, in several traits, such as tiller number, panicle shape, spikelet shape and fragility etc., the hybrids showed more resemblance to the wild tetraploid paternal species than to the maternal parent (Nipponbare) (**Fig 1C**), as expected given the 2:1 subgenome contributions to each hybrid by the two parental species for each triploid hybrid. All three hybrids are vigorous and can be maintained asexually by regeneration from nod-cuttings.

### All three sets of F1 triploid hybrids showed stochastic genetic and epigenetic changes at the molecular level

**Fig 2:**
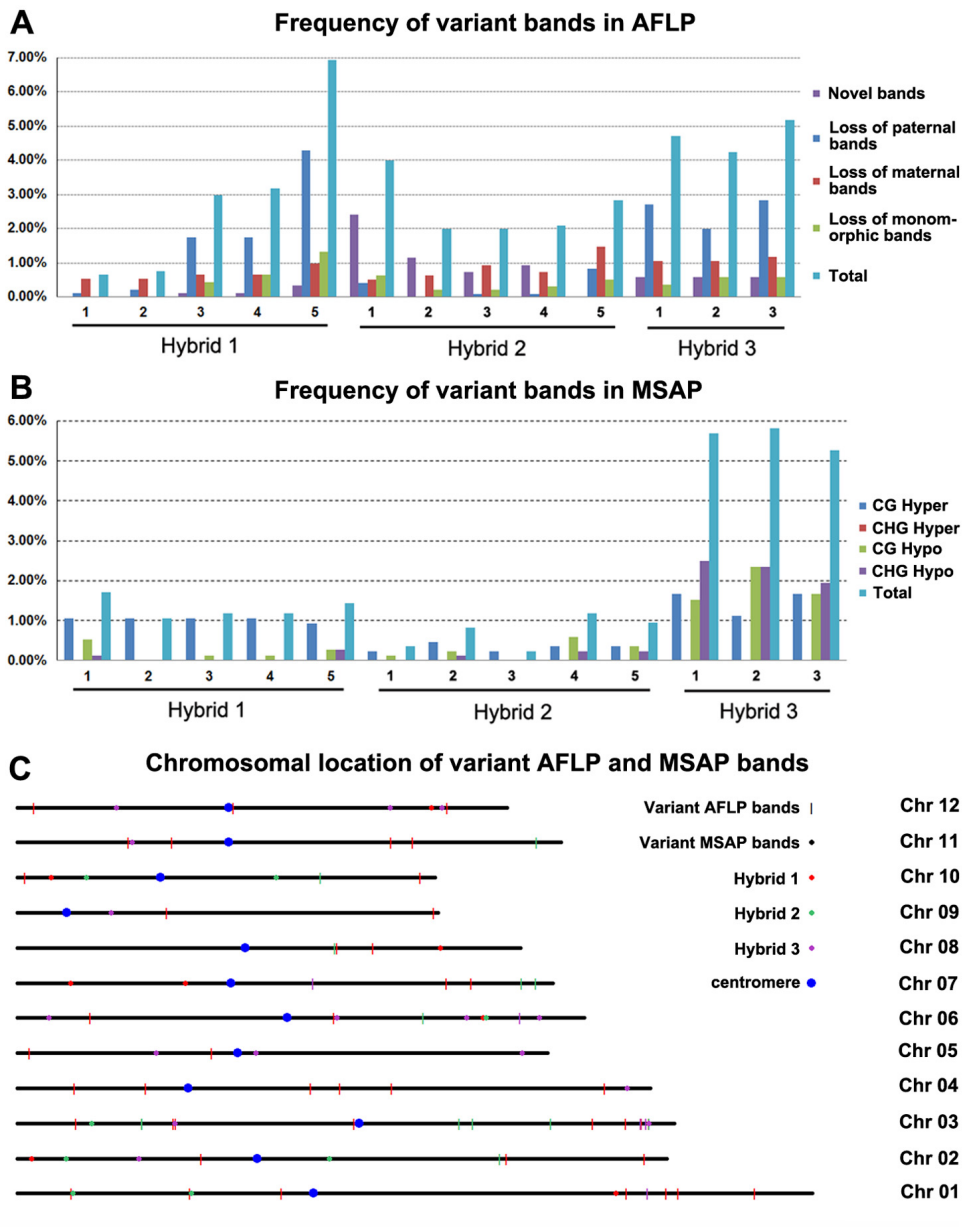
Frequencies and chromosomal location of genetic and DNA methylation changes detected by AFLP and MSAP. (A) Frequencies of genetic changes detected by AFLP analysis and revealed by various patterns of variant bands in the three F1 hybrids. (B) Frequencies of DNA methylation changes detected by MSAP analysis and revealed by various patterns of cytosine methylation in the three F1 hybrids. In both (A) and (B), the variant bands in the hybrids were determined by comparison with the parental mix. (C) Chromosomal location of the variant AFLP and MSAP bands. The three colors (red, green and purple) denote origins of variant bands from Hybrid 1, Hybrid 2 and Hybrid 3, respectively.

To explore whether genetic and epigenetic instability occurred immediately in somatic cells of the triploid hybrids as a result of merging of the divergent genomes, we conducted global analysis by using the AFLP and MSAP markers, respectively, which assay randomly targeted loci (harboring restriction sites of the enzymes used) across the hybrid genomes. For AFLP analysis, by using 18 pairs of selective primers (**S1 Table**), we scored 909, 952 and 849 loci for the three hybrids, respectively. All three hybrids showed nonadditivity in the banding patterns relative to their corresponding *in vitro* “hybrids”, indicating occurrence of genetic instabilities (**Fig 2A**). However, the extents of genetic instability varied markedly among the randomly chosen individuals within a given F1 hybrid as well as between the different hybrids (**Fig 2A**), pointing to the largely stochastic nature of the changes. For example, the collective (adding up all types) changes of the five individuals of hybrid 1 ranged from 0.7% to 6.9%, those of the five individuals of hybrid 2 ranged from 2.0% to 4.0%, and those of the three individuals of hybrid 3 ranged from 4.2% to 5.2% (**Fig 2A**). Notably, each of the four specific types of changes also varied substantially across the individuals within a hybrid and among the three hybrids; for example, while most of the band losses in hybrids 1 and 3 are from the paternal parent, those of hybrid 2 are mainly from the maternal parent (**Fig 2A**). In addition, more gains of new bands than loss of parental bands occurred in hybrids 2 and 3 than in hybrid 1 (**Fig 2A**).

The same plants analyzed by AFLP were subjected to MSAP analysis to test for occurrence and extent of DNA methylation changes at randomly sampled 5’-CCGG sites in the three F1 hybrids. By using 16 pairs of selective primers (**S1 Table**), we scored 762, 850, 722 loci for the three hybrids, respectively. To avoid mistakenly assigning genetic changes as cytosine methylation alterations in the MSAP data, we excluded loci that appeared or disappeared *simultaneously* (relative to the *in vitro* "hybrids") in both enzyme digests because which were more likely due to mutations of the 5'CCGG sites than methylation alterations [32]. We found that similar to the situation of genetic variation (**Fig 2A**), nonadditivity in the MSAP banding patterns were observed in all three hybrids (**Fig 2B**). Notably, apparently lower levels of DNA methylation changes than genetic changes were detected in hybrids 1 and 2; however, hybrid 3 showed a higher level of methylation changes than genetic changes (**Fig 2B**). Moreover, the four methylation changing patterns also varied dramatically among the three hybrids (**Fig 2B**). Interestingly, unlike the substantial among-individual differences within a given hybrid for genetic changes detected by AFLP (**Fig 2A**), the methylation changes are highly similar among the individuals within a hybrid (**Fig 2B**). Finally, hybrid 3 showed the highest frequencies of methylation changes (**Fig 2B**), although it is not particularly different from the other two hybrids in terms of genetic changes according to the AFLP data (**Fig 2A**). Among the four methylation patterns, CHG hyper-methylation was not detected, the rest three patterns, CG hyper-, CG hypo- and CHG hypo-methylation, all occurred but at variable frequencies (**Fig 2B**).

To gain insight into the chromosomal distribution and possible functional relevance of the genomic loci that showed genetic and epigenetic variations in the three hybrids, a randomly chosen subset of variable AFLP and MSAP bands were isolated, cloned and sequenced. It was found that both the AFLP and MSAP variable bands were mapped to all 12 rice chromosomes without notable mapping preference to a particular region or regions (**Fig 2C**). BlastN and BlastX analyses indicated that 50% and 54% sequenced variant bands were protein-coding sequences for AFLP and MSAP respectively, including known-functional protein-coding genes (28.0% and 28.1%), hypothetical protein-coding genes (5.3% and 20.2%) and TEs (**S2 Table**). The fact that *ca*. 50% genetic and DNA methylation-altered loci are landed in genic regions suggests that the rapid genomic changes are functionally relevant and may at least in part explain the emergent novel phenotypes of the hybrids (**Fig 1**). No correlation was detected between the AFLP and MSAP data (by Pearson correlation test) indicating largely independent occurrence of the genetic and epigenetic changes.

### Genomic regions adjacent to different TEs have differential stability in DNA methylation in the triploid hybrids

The foregoing results indicated that, overall, lower frequencies of DNA methylation changes than genetic changes occurred in two of the three hybrids (**Fig 2**). This is unexpected given that epigenetic states including DNA methylation are usually more prone to change than the primary nucleotide sequence as a result of stress like hybridization-induced genome shock [14, 16, 35]. Nevertheless, it is known that DNA methylation is unevenly distributed across plant genomes, with heterochromatic regions, being predominantly constituted by high copy-number TEs and their derivatives are more heavily methylated than euchromatic regions that are mainly comprised of low-copy genic sequences [36–40]. Therefore, it is possible that random sampling by the MSAP marker may result in underestimation of changes in DNA methylation at particular genomic regions if this kind of epigenetic changes occurred non-randomly across the hybrid genomes. To test this possibility, we analyzed methylation changes targeting to genomic regions flanking two previously characterized TEs of the rice genome, *mPing*, a relatively lower-copy number TE (*ca*. 50 copies) known to reside at low-copy genic regions [41, 42] and *Dasheng*, *a* highly reiterated TE (> 1,000 copies) known to reside at heterochromatin regions [43, 44]. We conducted the analysis by the element-specific molecular marker, termed methylation-sensitive transposon display (MSTD) [10], in the same sets of plants.

**Fig 3:**
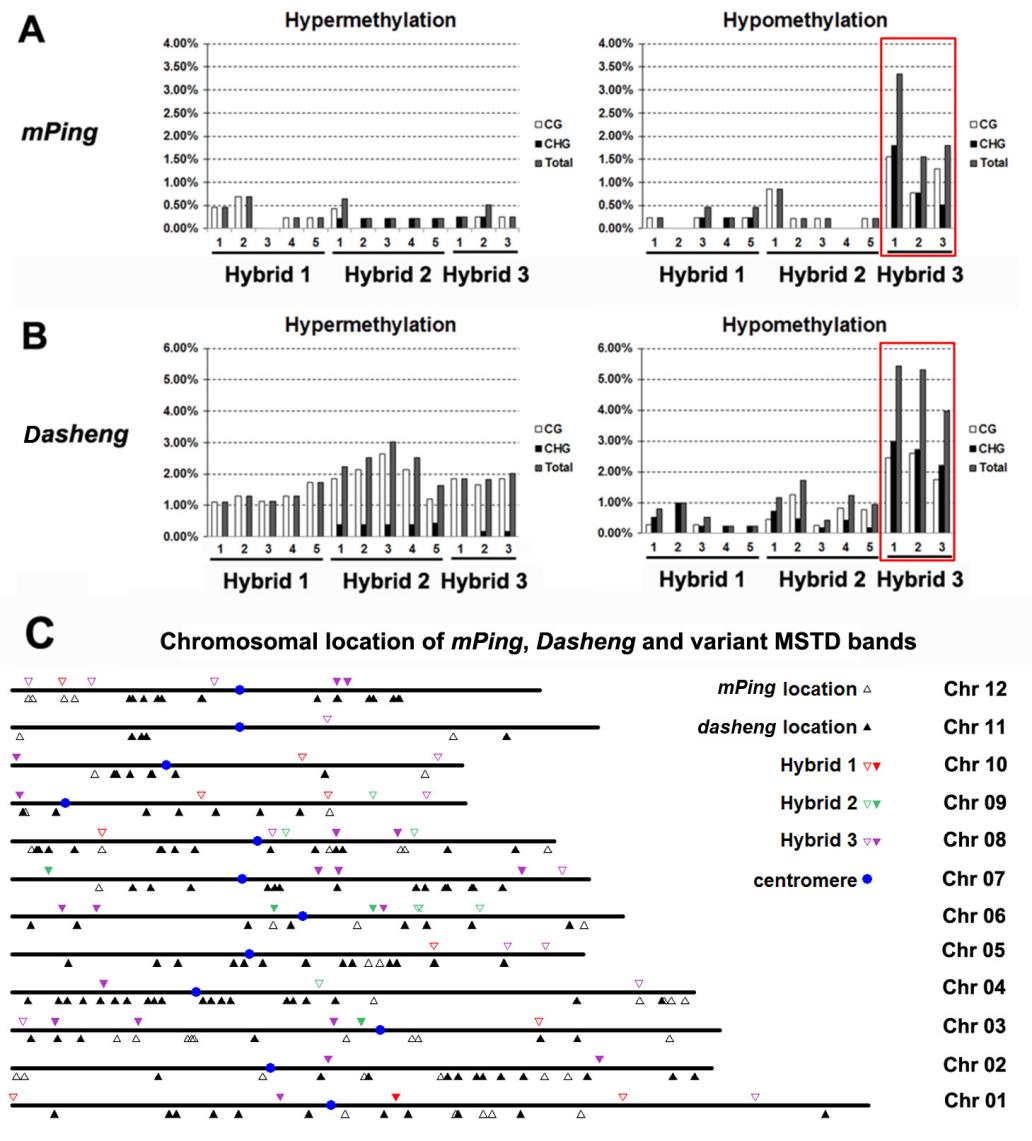
Frequencies and chromosomal locations of DNA methylation changes occurred in *mPing* and *Dasheng* flanking sequence. (A) Frequencies of DNA methylation changes in the 5’ flanks of *mPing* detected by MSTD in the three F1 hybrids. (B) Frequencies of DNA methylation changes in the 3’ flanks of *Dasheng* detected by MSTD in the three F1 hybrids. (C) Chromosomal locations of *mPing*, *Dasheng* innate copies in the Nipponbare genome and variant MSTD bands in the three F1 hybrids. Horizontal lines represent chromsomes of rice, triangles below the horizontal lines indicate the innate *mPing* and *Dasheng* copies, and triangles above the horizontal lines indicate the locations of DNA methylation changes.

The MSTD results showed that the total frequencies of methylation changes at genomic regions flanking *mPing* ranged from 0.5% to 0.7%, 0.2% to 1.5% and 2.1% to 3.6% in the three hybrids, 1, 2 and 3, respectively (**Fig 3A**), and which are similar (hybrid 2, *p* = 0.73 by Student’s t-test) to or even significantly lower (hybrids 1 and hybrids 3, both *p*<0.01 by Student’s t-test) than those revealed by MSAP, depending on the hybrids (**Fig 2B**). In contrast, the *Dasheng*-based MSTD results showed that the total frequencies of DNA methylation changes ranged from 1.5% to 2.3%, 2.6% to 4.3% and 6.0% to 7.3% in the three hybrids, respectively (**Fig 3B**), which are markedly higher than those revealed by MSAP, especially for hybrids 2 and 3 (*p* = 0.01, 0.00003 and 0.05 for hybrids 1, 2 and 3, respectively, by Student’s t-test)(**Fig 2B**). Together, the MSTD data suggest that genomic regions adjacent to different TEs have differential stability in DNA methylation when experiencing hybridization-invoked genetic stress: while low-copy genomic regions adjacent to *mPing* did not show an increased methylation instability than random loci sampled by the MSAP marker, high-copy heterochromatic regions flanking *Dasheng* showed significantly higher frequencies of methylation changes than those detected by MSAP. This observation is consistent with the known functions of DNA methylation as a repressive epigenetic marker for controlling heterochromatic TE activity [**45**]. Regarding the four methylation patterns, the three hybrids also showed variable results for genomic regions adjacent to the two TEs (**Fig 3A and 3B**). A striking similarity between the MSTD and the MSAP data is that hybrid 3 showed the highest frequencies of DNA methylation changes than hybrids 1 and 2 (**Figs 2B and 3A and 3B**). Notably, all the *mPing* and *Dasheng* endogenous loci were determined based on the Nipponbare genome reference (MSU7.0), and those methylation changes that located to regions outside of the *mPing*/*Dasheng* loci (**Fig 3C**) may have been from the paternal species (wild rice) whose genomes are largely unknown.

To test if there is any transpositional activation of these two TEs, we performed transposon-display (TD). Although nonadditive banding patterns were observed at frequencies similar to those detected by AFLP, none of a subset of nonadditive TD bands sequenced mapped to new genomic locations denoting for insertion events by the two TEs, suggesting the changed banding patterns in TD is not due to transposition of the TEs but to nucleotide changes, as in AFLP. However, we need to point out that the TD-based analysis cannot be considered as exhaustive to reveal low-abundance insertions.

### Transpositional and transcriptional activities of TEs and their relationship with methylation changes in the triploid hybrids

**Fig 4:**
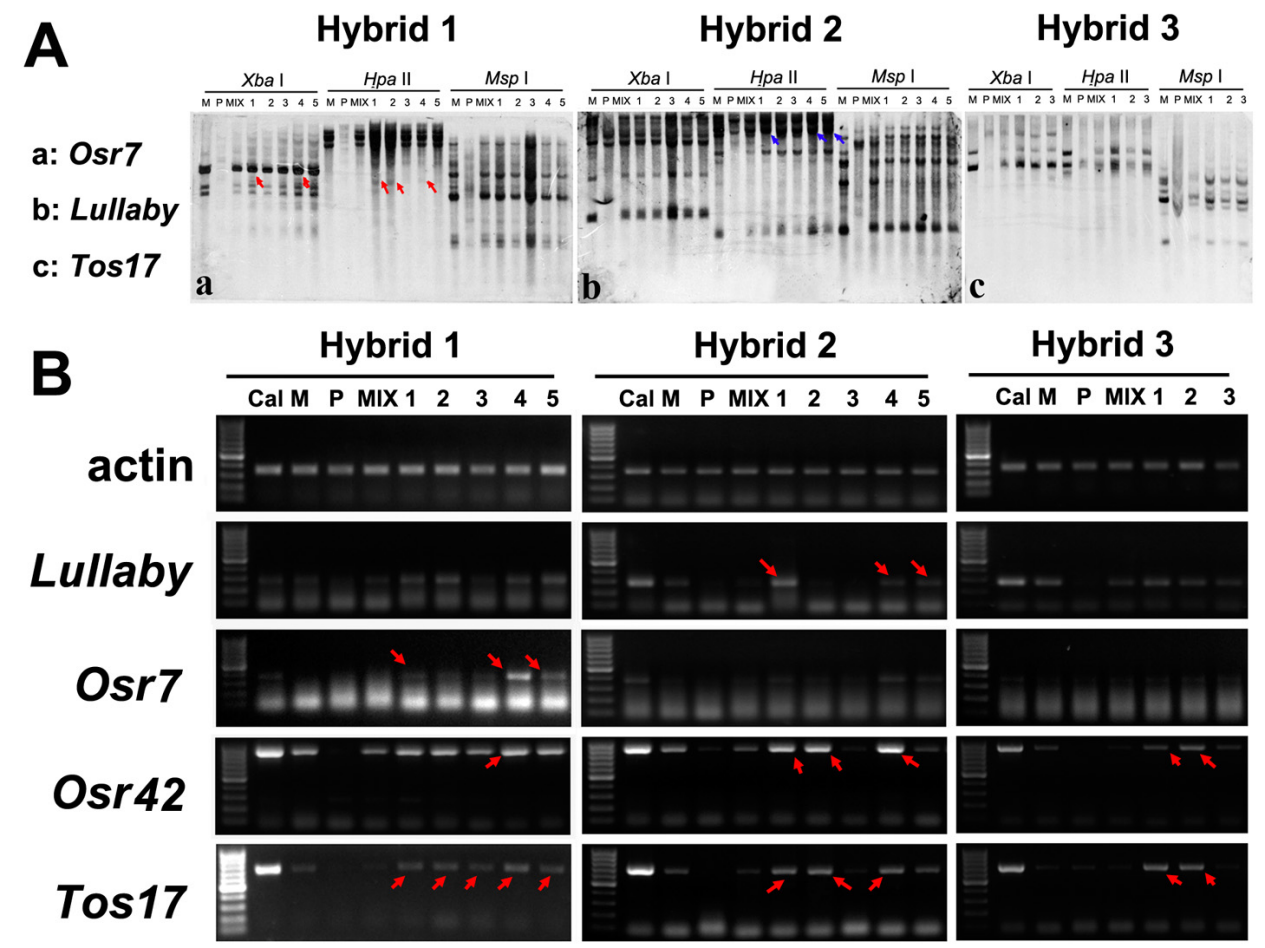
Examples of TE mobility, DNA methylation alterations, and transcriptional activation in the three F1 hybrids. (A) Southern blot hybridization patterns of three TEs in the three hybrids, respectively, along with the corresponding parents and their mix. The DNA samples were digested with *Xba*I and a pair of cytosine methylation-sensitive isoschizomers, *Hpa*II and *Msp*I, respectively. (a) *Osr7*, which showed transpositional activation in several individuals of Hybrid 1; (b) *Lullaby*, which showed CG hypomethylation in several individuals of Hybrid 2; (c) *Tos17*, which showed immobility in all individuals of Hybrid 3. Red arrow indicates novel bands, blue arrow indicates loss of bands. (B) Transcriptional activation of TEs in the three F1 hybrids based on semi-quentitative RT-PCR analysis. A cDNA sample from callus of Nipponbare (six-month old) was included as a positive control, red arrows indicate transcriptional activation in the hybrids relative to their parents and mix (the *invitro* "hybrids").

Several studies have reported that merging of divergent genomes by interspecific hybridization can induce transcriptional and/or transpositional activation of otherwise quiescent TEs of parental genomes, which often correlates with DNA methylation changes [5, 10, 11, 46]. To assess whether the stability of TEs would be immediately disrupted in these triploid hybrids, we chose 13 TEs that are known to possess transpositional potential under certain circumstances [45] for Southern blotting. The studied TEs included the 430 bp *mPing* [41, 42], its two transposase donors, *Ping* and *Pong* [41, 47], and 10 low-copy LTR retrotransposons, *Osr2*, *Osr3*, *Osr7*, *Osr23*, *Osr35*, *Osr36*, *Osr42*, *Tos19*, *Tos17* [48, 49] and *Lullaby* [50]. We used the same set of DNA samples and employed *Xba* I and a pair of methylation-sensitive isoschizomers, *Hpa*II and *Msp*I, to simultaneously assess copy number variation and cytosine methylation states of these TEs in the three hybrids relative to their parental plants. We then used RT-PCR to analyze possible transcriptional activation of these TEs in the hybrids relative to their parental plants. Southern blotting indicated that (1) at least two of the five studied plants of hybrid 1 showed increased copy numbers of two LTR retrotranspons, *Osr7* and *Osr36*, denoting their transpositional activation (e.g., **Fig 4A-a; Table 1**); (2) concomitant with (1), CG hypomethylation at the 5'CCGG sites (recognized by *Hpa*II) occurred in several TEs in most plants of hybrid 1 (e.g., **Fig 4A-a; Table 1**); (3) no change of copy number was detected for any of the TEs in hybrid 2, however, both hypo- and hypermethylation occurred in several plants of this hybrid (e.g., **Fig 4A-b**; **Table 1**); (4) changes in neither copy number nor methylation was detected in any of the TEs in hybrid 3 (e.g., **Fig 4A-c; Table 1**). Taken together, it appears that both transpositional activation of TEs and their methylation changes occurred in a genetic context-dependent manner, the two types of changes may or may not be coupled with each other, suggesting changes in DNA methylation or the extent thereof is likely required but not sufficient to cause TE mobility, consistent with previous findings [15, 51, 52].

**Table 1:** Transpositional activation and methylation changes in a set of transposable elements (TEs) in the three F1 hybrids of *Oryza*.

Probe		Alteration in copy number or DNA methylation pattern of TEs in interspecific hybrids of *Oryza* ^b^								
	Genbank accession^a^	Hybrid 1 (5 individuals)			Hybrid 2 (5 individuals)			Hybrid 3 (3 individuals)		
		*Xba* I	*Hpa* II	*Msp* I	*Xba* I	*Hpa* II	*Msp* I	*Xba* I	*Hpa* II	*Msp* I
*mPing*	AP005628	-	-	-	-	-	-	-	-	-
*Ping*	AB087616	-	-	-	-	-	-	-	-	-
*Pong*	BK000586	-	-	-	-	-	-	-	-	-
*Tos17*	AC087545	-	-	-	-	-	-	-	-	-
*Tos19*	T03721	-	-	-	-	-	-	-	-	-
*Lullaby*	AP008212	-	3 ↓	-	-	3 ↓	-	-	-	-
*Osr2*	AL442110	-	-	-	-	-	-	-	-	-
*Osr3*	AF458765	-	-	-	-	-	-	-	-	-
*Osr7*	AP002538	4 ↑	5 ↓	-	-	-	-	-	-	-
*Osr23*	AP002843	-	-	-	-	2 ↑	-	-	-	-
*Osr35*	AC068924	-	-	-	-	-	-	-	-	-
*Osr36*	AP001551	5 ↑	5 ↓	1 ↓	-	-	-	-	-	-
*Osr42*	AF458768	-	-	-	-	-	-	-	-	-

Note

^a^Determined by BlastN at NCBI

^b^Changes in copy number or DNA methylation pattern of TEs are defined as: “-”, No change; Arabic number, Number of changing individuals; “↑” in *Xba*I, Gained new copies in this hybrid group; “↑” in *Hpa*II and *Msp*I, Hypermethylation in the corresponding methylation sensitive restriction enzyme; “↓” in *Hpa*II and *Msp*I, Hypomethylation in the corresponding methylation sensitive restriction enzyme.

It should be noted that these F1 plants did not undergo meiosis, therefore all transpostional and methylation were somatic events. As such, if the changes did not occur early enough, then only a fraction of the somatic cells would contain the changes, and which may not be detectable by Southern blotting (e.g., **Fig 4A**), but can be detected by the PCR-based MSTD method (**Fig 3**). Therefore, both the transpostional and methylation changes detected by Southern blotting (**Table 1**) should be underestimates.

RT-PCR analysis showed that plants of all three hybrids showed transcriptional activation in at least some of the 12 studied TEs. Specifically, six TEs (*Ping*, *Lullaby*, *Osr3*, *Osr7*, *Osr42* and *Tos17*) showed activation in several plants of hybrid 1, four TEs (*Lullaby*, *Tos17*, *Osr36* and *Osr42*) showed activation in several plants of hybrid 2, and six TEs (*Ping*, *Osr2*, *Osr3*, *Osr7*, *Osr35* and *Tos17*) showed activation in hybrid 3 (e.g., **Fig 4B; Table 2**). This suggests that transcriptional activation of TEs occurred more generally in these hybrid plants, which is not necessarily correlating with methylation alterations. However, we cannot rule out the possibility that a correlation may emerge if the methylation was analyzed at the base pair resolution.

**Table 2:** Transcriptional activation on a set of transposable elements (TEs) in three triploid F1 hybrids of *Oryza*.

Probe		Alteration in expression level of TEs in the F1 hybrids ^b^												
	Genbank accession ^a^	Hybrid 1 (5 individuals)					Hybrid 2 (5 individuals)					Hybrid 3 (3 individuals)		
		1	2	3	4	5	1	2	3	4	5	1	2	3
*Ping*	AB087616	↑	↑	↑	↑	↑	-	-	-	-	-	↑	↑	↑
*Pong*	BK000586	↑	↑	-	-	-	-	-	-	-	-	-	-	-
*Tos17*	AC087545	↑	↑	↑	↑	-	↑	↑	↑	↑	↑	↑	↑	-
*Lullaby*	AP008212	↑	↑	-	↑	↑	↑	-	-	↑	↑	-	-	-
*Osr2*	AL442110	-	-	↑	-	↑	-	-	-	-	-	↑	-	↑
*Osr3*	AF458765	-	↑	↑	↑	↑	-	-	-	-	-	↑	↑	↑
*Osr7*	AP002538	↑	↑	↑	↑	↑	-	-	-	-	-	-	-	↑
*Osr23*	AP002843	-	-	-	-	-	-	-	-	-	-	-	-	-
*Osr35*	AC068924	↑	-	-	-	↓	-	-	-	-	-	↑	↑	-
*Osr36*	AP001551	-	-	-	-	-	-	-	↑	-	-	-	-	-
*Osr42*	AF458768	↑	↑	↑	↑	↑	↑	↑	↑	↑	↑	↑	↑	-
Freq. (%)		63.6	54.5	54.5	54.5	63.6	27.3	18.2	27.3	27.3	27.3	54.5	45.5	36.4

Note

^a^ Determined by BlastN at NCBI

^b^ Changes in expression levels of TEs are defined as: “-”, No change; “↑”, Expression level being significantly increased; “↓”, Expression level being significantly decreased.

## Discussion

Interspecific hybridization, being frequent and recurring, is a rich source of genetic variation underlying phenotypic novelty in the resulting F1 hybrids [4]. It is widely known that hybrids often show phenotypes that are not merely the additivity or intermediacy of their parents, as collectively best illustrated by the phenomenon of hybrid vigor or heterosis. The phenotypic novelty of F1 hybrids may have been caused by multiple causes and their myriad interactions, which include, but are not limited to, physiological responses, changes in gene expression, molecular level genetic and epigenetic changes, and karyotype repatterning [4]. We have reported recently that one of the triploid *Oryza* hybrids studied here (hybrid 2) showed significant changes of genome-wide total gene expression and homeolog expression partitioning, which may partly explain the changed phenotypes [34]. Here we show that all three sets of interspecific triploid hybrids are karyotypically stable (i.e., lack of chromosomal repatterning), and therefore, their altered gene expression and novel phenotypes should be largely due to molecular level interactions and *de novo* changes. Indeed, we detected variable frequencies of loss and gain of bands in the AFLP profiles of all the three hybrids studied. Because the maternal parent of all three sets of hybrids was the standard laboratory rice cultivar, Nipponbare, which is an artificially maintained pure line, and all three paternal wild rice species accessions had also been extensively selfed for at least five consecutive generations in our hands before the crosses were made, we can confidently rule out the possibility that the genetic changes detected in the hybrids are due to segregation of parental heterozygosity. Moreover, gain of novel bands, loss of maternal bands (Nipponbare) and loss of monomorphic bands cannot be explained by segregation of parental heterozygosity. We assume that nucleotide base changes, probably due to compromised repairing machinery in the hybrid genomes, occurred at the *Mse*I and/or *Eco*RI enzyme loci leading to restriction failure and/or gain (though which should be less likely). Additional causes for AFLP band loss in the hybrids might be due to TE-mediated physical elimination of sequences. Although recombination-mediated sequence loss should be mainly meiotic events [53], it was shown recently that sequence deletion may also occur in somatic cells of wheat-*Aegilops* allopolyploids [54] and F1 hybrids [55]. Remarkably, it was found that in some of these F1 hybrids loss of retrotransposon-related sequences was rampant and positively related to parental divergence and hence incompatibility of the retrotransposons [55].

It has been reported in diverse plant taxa that interspecific hybridization and allopolyploidization can induce extensive changes in DNA methylation, and which often occur to similar extents as, or more prevalent than, genetic changes [16]. For example, in naturally formed *Spartina* allotetraploids there were little genetic changes; by contrast, changes in DNA methylation occurred in > 30% loci assessed [14]. Also, in euploid individuals of several synthetic allohexaploid wheats (analogous to natural common wheat in genome constitution), rapid genetic changes were rare or nonexistent but DNA methylation repatterning occurred extensively [35]. Here, we found that both genetic changes and DNA methylation repatterning occurred immediately (without entailing meiosis) in all three sets of rice F1 hybrids harboring divergent subgenomes. Surprisingly, however, at least in two of the hybrids, more genetic than methylation changes were detected. The cause for this unexpected observation is unclear but it may, at least in part, be due to the uneven distribution of methylation changes across the rice genome. For example, more methylation changes in heterochromatic genomic regions flanking the high copy TE, *Dasheng* [43, 44] than the low copy genic regions adjacent to the low-copy TE, *mPing* [41, 42] were detected based on the TE-display results. Thus, in theory, the largely unbiased sampling by the MSAP marker [32] may underestimate the extent of methylation changes in the hybrids. Alternatively, our scoring strategy of the MSAP data [32] by excluding all loci that showed simultaneous alteration in the pair isoschizomers (*Hpa*II and *Msp*I) may also cause underestimation of DNA methylation changes. However, this should be a minor cause because in theory concordant changes in methylation at the two cytosine residues of the same 5'CCGG site) is much less likely than a single base change, given that methylation of CG and CHG is known to be maintained by distinct enzymes [38, 39].

Several studies on hybrids and allopolyploids have demonstrated that the merging of divergent genomes can cause transcriptional and transpositional reactivation of otherwise dormant parental TEs, which are often associated with changes in DNA methylation [5, 10, 11, 45, 56]. Here we found that of the 13 TEs analyzed by RT-PCR, 10 showed enhanced expression level in the F1 hybrids relative to their parents, suggest widespread transcriptional activation of TEs. In contrast, only two TEs showed increased copy number in some of the hybrid individuals denoting their transpositional activation. The lack of coupling between changes in DNA methylation, transcription and transposition of TEs in newly formed plant hybrids is consistent with previous observations. For example, in contemporary sunflower hybrids populations, widespread transcriptional activity of the LTR type of TEs was found but no evidence of their transposition was detected [57]. Similarly, although extensive DNA methylation changes occurred in genomic regions flanking TEs in hybrids and allopolyploids of *Spartina*, no activity of these TEs was detected [15].

A salient feature of our study system is that all three sets of hybrids share the same maternal parent (Nipponbare) but with different wild rice species as the paternal parents. Therefore, the differences among the sets in genetic and epigenetic instabilities can be explained by genetic context effects. However, the differences among the individuals within the same hybrid are apparently due to stochasticity. Indeed, stochastic genetic and epigenetic variations have been increasingly recognized as an important force driving evolution, especially under changing environments [58]. Therefore, merging of divergent genomes into one nucleus and cytoplasm via interspecific hybridization represents an efficient avenue to generate useful variations for natural and artificial selections to act upon. In our case, although the hybrids are male sterile, we noted that the female gametes developed normally, and therefore they might be used as maternal parents to be backcrossed with genotypes of cultivated rice and introduce the novel variations to the later.

## Materials and Methods

### Plant Materials and Growth Conditions

Three sets of interspecific rice hybrids were produced by crossing each of three tetraploid wild species of *Oryza* as paternal parents with the standard laboratory rice cultivar Nipponbare (*O*.*sativa* ssp. *japonica*, cv.) as the maternal parent were produced assisted by embryo rescue. Accessions of the three wild tetraploid rice species were *O*. *alta* (IRGC Acc. No. 100161, CCDD genome), *O*. *punctata* (IRGC Acc. No. 106304, BBCC genome), and *O*. *officinalis* (IRGC Acc. No. 100953, CCC’C’ genome), respectively and their original seeds were kindly provided by Dr. Song Ge at the Institute of Botany, Chinese Academy of Sciences. The accessions were extensively selfed for at least 5 consecutive generations before the crosses were made. Genomic DNA of two diploid wild rice species used to differentiate the wild tetraploid genomes in the genomic in situ hybridization (GISH) analysis were extracted from *O*. *punctata* (IRGC Acc. No. 104974, BB genome) and *O*. *officinalis* (IRGC Acc. No. 100953, CC genome), which were kindly provided by Dr. N.R. Sackville Hamilton at the International Rice Research Institute (IRRI). The three sets of F1 triploid hybrids produced were designated as hybrid 1 (genome constitution: ACD, between Nipponbare and *O*. *alta*), hybrid 2 (genome constitution: ABC, between Nipponbare and *O*. *punctata*), hybrid 3 (genome constitution: ACC’, between Nipponbare and *O*. *officinalis*). All the plants used in this study were planted in the greenhouse at 26±3°C with a 16 hr/8 hr (light/dark) photoperiod.

### Genomic in situ hybridization (GISH)

The protocol was essentially described [59] with minor modifications [60]. Specifically, genomic DNA was isolated from young leaves of Nipponbare and the two diploid wild rice species, *O*. *punctata* (BB genome), and *O*. *officinalis* (CC genome), by the CTAB method. Genomic DNA of Nipponbare was labeled by nick translation with Texas Red-5-dCTP (Perkin Elmer; cat. no. NEL 426). Genomic DNA of *O*. *punctata* and *O*. *officinalis* was labeled by nick translation with Chroma Tide Alexa Fluor 488-5-dUTP (Invitrogen; cat. no. C11397), respectively. Because of the difficulty to separate the C and D genomes in both *O*. *alta* and hybrid 1, to distinguish between them, we controlled the probe fragment length in the range of 400–500 bp as recommended in a previous study [61]. Slide denaturation, hybridization, and washing conditions were carried out per the manufacturer's recommendation (Invitrogen; cat. no. C11397). Slides were examined by using an Olympus fluorescence microscope and digitally photographed.

### Amplified fragment length polymorphism (AFLP) and methylation-sensitive amplified fragment length polymorphism (MSAP)

Three to five individual plants were chosen from each of the three F1 hybrids, 1, 2 and 3, along with their exact parental plants. Genomic DNA was extracted from expanded young leaves of these plants. The parental DNAs were mixed at a ratio of 1:2 (in quantity) according to their genome contributions to each of the three triploid hybrids, and designated the mixtures as *in vitro* “hybrids”. The protocols used for amplified fragment length polymorphism (AFLP) and methylation-sensitive amplified fragment (MSAP) analysis were exactly as reported [32]. The primers used were listed in S1 Table. Only clear and completely reproducible bands (between two technical replicates) were scored. Typical bands representing genetic changes (AFLP) and cytosine methylation alterations (MSAP) in the F1 hybrids *vs*. the corresponding *in vitro* “hybrids” were isolated, cloned and sequenced. Homology analysis was performed by BlastN and BlastX at the NCBI website (http://www.ncbi.nlm.nih.gov/blast/Blast.cgi). We used R software (R version 3.0.1) to analyze the chromosomal distribution of the variant loci in the rice genome.

### Transposon display (TD) and methylation-sensitive transposon display (MSTD)

The same plants analyzed by AFLP and MSAP were also used in transposon display (TD) and methylation sensitive transposon display (MSTD) analysis. The protocols for transposon display (TD) and methylation sensitive transposon display (MSTD) were essentially as reported (10). Nested primers respectively specific to one terminus of each of the two TEs, *mPing* and *Dasheng*, were designed. The *mPing* specific primer was designed in the the terminal of 5’ end including the inverted repeat while the *Dasheng*-specific primer was designed on its 3’ LTR. These TE-specific primers were coupled with a primer matching the *Mse*I adaptor sequence were used for amplifications. All primers used in TD and MSTD are listed in S3 Table. Only clear and completely reproducible bands (between two technical replicates) were scored. A subset of variant bands between the F1 hybrids and their corresponding *in vitro* “hybrids” was isolated, cloned and sequenced. Fragment location and homology analysis were performed as for those of AFLP and MSAP, described above.

### Southern blot analysis

The same genomic DNAs was used in Southern blot analysis. Conventional and methylation-sensitive gel-blotting followed the protocols described earlier [30, 51]. Genomic DNA (~6 μg per lane) was digested by each of the three enzymes *Xba*I, *Hpa*II and *Msp*I (purchased from New England Biolabs Inc. (Beverly, Massachusetts), and run through 1% agarose gels. *Xba*I was used for the purpose of copy number estimation of the TEs because all these elements did not have a restriction site or the site(s) being on one side of the probe region only. *Hpa*II and *Msp*I were used to assess possible DNA methylation changes within or adjacent to the TEs. Fractionated DNA was transferred onto Hybond N+ nylon membranes (Amersham Pharmacia Biotech) by the neutral transfer recommended by the supplier. In total, probe fragments representing a total of 13 low-copy TEs were amplified by PCR from genomic DNA of Nipponbare (S4 Table), and authenticity of the fragments as part of the TEs were confirmed by sequencing. The fragments were labeled with fluorescein-11-dUTP by the Gene Images random prime-labeling module (Amersham Pharmacia Biotech). Hybridization signal was detected by the Gene Images CDP-Star detection module (Amersham Pharmacia Biotech) after washing at a stringency of 0.2 × SSC, 0.1% SDS for 2 × 50 min. The filters were exposed to X-ray film for 1–3 hrs depending on signal intensity [30, 51].

### Reverse-transcription (RT) PCR analysis of TE transcriptional activity

The protocol was as reported [30]. Specifically, total RNA was isolated from expanded young leaves at the same developmental stage as used for DNA isolation by the Trizol Reagent (Invitrogen), following the manufacturer’s protocol. The RNA was treated with DNaseI (Invitrogen), reverse-transcribed by the SuperScript RNase H-Reverse Transcriptase (Invitrogen), and subjected to RT-PCR analysis using gene-specific primers. A rice actin gene (Genbank accession X79378) was used as a control for normalization of RNA input. The primers used for RT-PCR were listed in S5 Table. DNA contamination was tested by inclusion of RNAs without RT. For RT-PCR, amplification of cDNAs was performed for 30 cycles at an annealing temperature of 60°C. Three batches of independently prepared total RNAs were used as technical replications. The amplifications were visualized by ethidium bromide staining after electrophoresis through 1.5% agarose gels. Amplifications of the correct sizes were cloned for sequencing to verify their authenticity. For each run, a cDNA from callus of Nipponbare (six-month old) was used as a positive control, because it is known that most of these TEs were transcriptionally activated in callus [50]. We also used a DNA control in each run to ensure efficient amplification for each set of primers. Then we used the ImageJ software (http://imagej.nih.gov/ij/) to quantify the agarose gel electrophoresis images and normalized the expression levels of TEs to that of actin. We tested for differential expression of the TEs by Student’s t-test (assigned as significant differential expression if *p*<0.01; otherwise as no change).

## Supporting Information

S1 Table AFLP and MSAP adapters/primers used. (DOC)

S2 Table BlastN and BlastX analyses for sequenced variant bands in AFLP and MSAP. (DOC)

S3 Table TD and MSTD adapters/primers used. (DOC)

S4 Table The list of primers of probes used in Southern-blotting. (DOC)

S5 Table RT-PCR primers used. (DOC)
