# Supplementary material for: Immediate Genetic and Epigenetic Changes in F1 Hybrids Parented by Species with Divergent Genomes in the Rice Genus (Oryza)
Source: PLoS One. 2015 Jul 24;10(7):e0132911. doi: 10.1371/journal.pone.0132911 (PMC4514751; doi:10.1371/journal.pone.0132911)
Supplement: S1 Table — (DOC) [file pone.0132911.s001.doc]

**Table S1.** AFLP and MSAP adapters/primers used in this study

| **Adapter and primers used in AFLP** | | | **Adapter and primers used in MSAP** | | |
| --- | --- | --- | --- | --- | --- |
| Name | Sequence | Sequence | Name | Sequence | Sequence |
| *Mse*I adapterI | 5’-GACGATGAGTCCTGAG-3’ |  | H/M adapterI | 5’-GATCATGAGTCCTGCT-3’ |  |
| *Mse*I adapterII | 5’-TACTCAGGACTCAT-3’ |  | H/M adapterII | 5'-CGAGCAGGACTCATGA-3’ |  |
| *Eco*RI adapterI | 5'-CTCGTAGACTGCGTACC-3’ |  | *Eco*RⅠI adapterI | 5'-CTCGTAGACTGCGTACC-3’ |  |
| *Eco*RI adapterII | 5'-AATTGGTACGCAGTC-3’ |  | *Eco*RⅠI adapterII | 5'-AATTGGTACGCAGTC-3’ |  |
| Pre-selective primers |  |  | Pre-selective primers |  |  |
| *Mse*I+ C | 5’-GATGAGTCCTGAGTAAC-3’ |  | H/M + T | 5’-ATCATGAGTCCTGCTCGGT-3’ |  |
| *Eco*RI+ A | 5’-GACTGCGTACCAATTCA-3’ |  | *Eco*RI+ A | 5’-GACTGCGTACCAATTCA-3’ |  |
| Selective primers | *Eco*RI+3 (A-I) | *Mse*I+3 (1-9) | Selective primers | *Eco*RI+3 (A-D) | *Hpa*II+3 (2-10) |
| A5 | 5’-GACTGCGTACCAATTCAAC-3’ | 5’-GATGAGTCCTGAGTAACTA-3’ | A5 | 5’-GACTGCGTACCAATTCAAC-3’ | 5'-ATCATGAGTCCTGCTCGGTTG-3’ |
| B4 | 5’-GACTGCGTACCAATTCAAG-3’ | 5’-GATGAGTCCTGAGTAACAT-3’ | A7 | 5’-GACTGCGTACCAATTCAAC-3’ | 5'-ATCATGAGTCCTGCTCGGTGA-3’ |
| B5 | 5’-GACTGCGTACCAATTCAAG-3’ | 5’-GATGAGTCCTGAGTAACTA-3’ | A9 | 5’-GACTGCGTACCAATTCAAC-3’ | 5'-ATCATGAGTCCTGCTCGGTGC-3’ |
| B6 | 5’-GACTGCGTACCAATTCAAG-3’ | 5’-GATGAGTCCTGAGTAACTC-3’ | B4 | 5’-GACTGCGTACCAATTCAAG-3’ | 5'-ATCATGAGTCCTGCTCGGTTC-3’ |
| C5 | 5’-GACTGCGTACCAATTCACA-3’ | 5’-GATGAGTCCTGAGTAACTA-3’ | B5 | 5’-GACTGCGTACCAATTCAAG-3’ | 5'-ATCATGAGTCCTGCTCGGTTG-3’ |
| C7 | 5’-GACTGCGTACCAATTCACA-3’ | 5’-GATGAGTCCTGAGTAACTG-3’ | B6 | 5’-GACTGCGTACCAATTCAAG-3’ | 5'-ATCATGAGTCCTGCTCGGTTA-3’ |
| C9 | 5’-GACTGCGTACCAATTCACA-3’ | 5’-GATGAGTCCTGAGTAACCA-3’ | B8 | 5’-GACTGCGTACCAATTCAAG-3’ | 5'-ATCATGAGTCCTGCTCGGTGT-3’ |
| D4 | 5’-GACTGCGTACCAATTCACT-3’ | 5’-GATGAGTCCTGAGTAACAT-3’ | C2 | 5’-GACTGCGTACCAATTCACA-3’ | 5'-ATCATGAGTCCTGCTCGGTCG-3’ |
| D5 | 5’-GACTGCGTACCAATTCACT-3’ | 5’-GATGAGTCCTGAGTAACTA-3’ | C5 | 5’-GACTGCGTACCAATTCACA-3’ | 5'-ATCATGAGTCCTGCTCGGTTG-3’ |
| D6 | 5’-GACTGCGTACCAATTCACT-3’ | 5’-GATGAGTCCTGAGTAACTC-3’ | C9 | 5’-GACTGCGTACCAATTCACA-3’ | 5'-ATCATGAGTCCTGCTCGGTGC-3’ |
| D7 | 5’-GACTGCGTACCAATTCACT-3’ | 5’-GATGAGTCCTGAGTAACTG-3’ | D4 | 5’-GACTGCGTACCAATTCACT-3’ | 5'-ATCATGAGTCCTGCTCGGTTC-3’ |
| D9 | 5’-GACTGCGTACCAATTCACT-3’ | 5’-GATGAGTCCTGAGTAACCA-3’ | D5 | 5’-GACTGCGTACCAATTCACT-3’ | 5'-ATCATGAGTCCTGCTCGGTTG-3’ |
| E5 | 5’-GACTGCGTACCAATTCACT-3’ | 5’-GATGAGTCCTGAGTAACTA-3’ | D7 | 5’-GACTGCGTACCAATTCACT-3’ | 5'-ATCATGAGTCCTGCTCGGTGA-3’ |
| E7 | 5’-GACTGCGTACCAATTCACT-3’ | 5’-GATGAGTCCTGAGTAACTG-3’ | D8 | 5’-GACTGCGTACCAATTCACT-3’ | 5'-ATCATGAGTCCTGCTCGGTGT-3’ |
| F5 | 5’-GACTGCGTACCAATTCACG-3’ | 5’-GATGAGTCCTGAGTAACTA-3’ | D9 | 5’-GACTGCGTACCAATTCACT-3’ | 5'-ATCATGAGTCCTGCTCGGTGC-3’ |
| F9 | 5’-GACTGCGTACCAATTCACG-3’ | 5’-GATGAGTCCTGAGTAACCA-3’ | D10 | 5’-GACTGCGTACCAATTCACT-3’ | 5'-ATCATGAGTCCTGCTCGGTAC-3’ |
| H5 | 5’-GACTGCGTACCAATTCAGG-3’ | 5’-GATGAGTCCTGAGTAACTA-3’ |  |  |  |
| I5 | 5’-GACTGCGTACCAATTCAGA-3’ | 5’-GATGAGTCCTGAGTAACTA-3’ |  |  |  |
