# Supplementary material for: Immediate Genetic and Epigenetic Changes in F1 Hybrids Parented by Species with Divergent Genomes in the Rice Genus (Oryza)
Source: PLoS One. 2015 Jul 24;10(7):e0132911. doi: 10.1371/journal.pone.0132911 (PMC4514751; doi:10.1371/journal.pone.0132911)
Supplement: S2 Table — (DOC) [file pone.0132911.s002.doc]

**Table S2.** BlastN and BlastX analyses for sequenced nonadditive bands in AFLP and MSAP

| **Marker** | **Category** | **number** | **percentage** |
| --- | --- | --- | --- |
| AFLP | TEs | 22 | 16.67% |
|  | functional protein | 37 | 28.03% |
|  | hypothetical protein | 7 | 5.30% |
|  | intergenic | 26 | 19.70% |
|  | No homology | 40 | 30.30% |
|  | Total | 132 | 100.00% |
| MSAP | TEs | 5 | 5.62% |
|  | functional protein | 25 | 28.09% |
|  | hypothetical protein | 18 | 20.22% |
|  | intergenic | 24 | 26.97% |
|  | No homology | 17 | 19.10% |
|  | Total | 89 | 100.00% |
