# Supplementary material for: Immediate Genetic and Epigenetic Changes in F1 Hybrids Parented by Species with Divergent Genomes in the Rice Genus (Oryza)
Source: PLoS One. 2015 Jul 24;10(7):e0132911. doi: 10.1371/journal.pone.0132911 (PMC4514751; doi:10.1371/journal.pone.0132911)
Supplement: S3 Table — (DOC) [file pone.0132911.s003.doc]

**Table S3.** TD and MSTD adapters/primers used in this study

| **Adapter and primers used in TD** | | | **Adapter and primers used in MSTD** | |
| --- | --- | --- | --- | --- |
| Name | Sequence | Name | | Sequence |
| *Mse*I adapterI | 5’-GACGATGAGTCCTGAG-3’ | H/M adapterI | | 5’-GATCATGAGTCCTGCT-3’ |
| *Mse*I adapterII | 5’-TACTCAGGACTCAT-3’ | H/M adapterII | | 5'-CGAGCAGGACTCATGA-3’ |
| Pre-selective primers |  | Pre-selective  primers | |  |
| (*Mse*I+0) | 5’-GATGAGTCCTGAGTAAC-3’ | (H/M+0) | | 5’-ATCATGAGTCCTGCTCGG-3’ |
| *mPing* 5’-specific  primer | 5’-GCTGACGAGTTTCACCAGGAT-3’ | *mPing* 5’-specific  primer | | 5’-GCTGACGAGTTTCACCAGGAT-3’ |
| Selective primers  (*Mse*I+3) |  | Selective primers  (H/M+3) | |  |
| *Mse*I 1 | 5’-GATGAGTCCTGAGTAAAA-3’ | H/M 1 | | 5'-ATCATGAGTCCTGCTCGGAA-3’ |
| *Mse*I 2 | 5’-GATGAGTCCTGAGTAAAT-3’ | H/M 2 | | 5'-ATCATGAGTCCTGCTCGGAT-3’ |
| *Mse*I 3 | 5’-GATGAGTCCTGAGTAAAC-3’ | H/M 3 | | 5'-ATCATGAGTCCTGCTCGGAC-3’ |
| *Mse*I 4 | 5’-GATGAGTCCTGAGTAAAG-3’ | H/M 4 | | 5'-ATCATGAGTCCTGCTCGGAG-3’ |
| *Mse*I 5 | 5’-GATGAGTCCTGAGTAAGA-3’ | H/M 5 | | 5'-ATCATGAGTCCTGCTCGGGA-3’ |
| *Mse*I 6 | 5’-GATGAGTCCTGAGTAAGC-3’ | H/M 6 | | 5'-ATCATGAGTCCTGCTCGGGC-3’ |
| *Mse*I 7 | 5’-GATGAGTCCTGAGTAAGT-3’ | H/M 7 | | 5'-ATCATGAGTCCTGCTCGGGT-3’ |
| *Mse*Ⅰ 8 | 5’-GATGAGTCCTGAGTAAGC-3’ | H/M 8 | | 5'-ATCATGAGTCCTGCTCGGGG3’ |
| *Mse*Ⅰ 9 | 5’-GATGAGTCCTGAGTAATA-3’ | H/M 9 | | 5'-ATCATGAGTCCTGCTCGGTA-3’ |
| *Mse*Ⅰ 10 | 5’-GATGAGTCCTGAGTAATT-3’ | H/M 10 | | 5'-ATCATGAGTCCTGCTCGGTT-3’ |
| *Mse*Ⅰ 11 | 5’-GATGAGTCCTGAGTAATG-3’ | H/M 11 | | 5'-ATCATGAGTCCTGCTCGGTG-3’ |
| *Mse*Ⅰ 12 | 5’-GATGAGTCCTGAGTAATC-3’ | H/M 12 | | 5'-ATCATGAGTCCTGCTCGGTC-3’ |
| *mPing* 5’-specific  primer | 5’-TGTGCATGACACACCAGTG-3’ | *mPing* 5’-specific  primer | | 5’-TGTGCATGACACACCAGTG-3’ |
|  | (combined with the *Mse*ⅠSelective primers) |  |  | (combined with the H/M Selective primers) |
| *Dasheng* 3’-specific  primer | 5’-TTTAGGTCTCGTGCGCTACC-3’ | *Dasheng* 3’-specific  primer | | 5’-TTTAGGTCTCGTGCGCTACC-3’ |
|  | (combined with the *Mse*ⅠSelective primers) |  |  | (combined with the H/M Selective primers) |
