# Supplementary material for: Immediate Genetic and Epigenetic Changes in F1 Hybrids Parented by Species with Divergent Genomes in the Rice Genus (Oryza)
Source: PLoS One. 2015 Jul 24;10(7):e0132911. doi: 10.1371/journal.pone.0132911 (PMC4514751; doi:10.1371/journal.pone.0132911)
Supplement: S4 Table — (DOC) [file pone.0132911.s004.doc]

**Table S4.** The list of primers of probes used in DNA gel-blotting analysis

| **Probe** | **Genbank accession** | **Forward primer Sequence** | **Reverse primer Sequence** |
| --- | --- | --- | --- |
| *mPing* | AP005628 | 5’-GTCACAATGGGGGTTTCACT-3’ | 5’-GGCCAGTCACAATGGCTAGT-3’ |
| *Pong* | BK000586 | 5’-GGGGTGAAACAGCATTGAGA-3’ | 5’-TGTGGTTGCAAAGAAGACCA-3’ |
| *Ping* | AB087616 | 5’-CTACGGAGTACACCGCAACC-3’ | 5’-AATGGATTGCCTACTGCTGACT-3’ |
| *Osr2* | AL442110 | 5’-CACACCAGCACCAAGTCCTA-3’ | 5’-TCGATCGCTTTAGGTTGCTT-3’ |
| *Osr3* | AF458765 | 5’-ATCGACATACAGGGCCTTTG-3’ | 5’-TCAGCAACTTGTCCACCAGA-3’ |
| *Osr7* | AP002538 | 5’-AGAGCCCGGTTAAGTTCGTT-3’ | 5’-AGCTTGTCCATGGTAAGGTCA-3’ |
| *Osr23* | AP002843 | 5’-GCCGGTCTTGATGATGAGTT-3’ | 5’-TTGAACAGACGCTCCACAAG-3’ |
| *Osr35* | AC068924 | 5’-TGATGTGGTCCTTGAGTCCA-3’ | 5’-ATTCTCTTGGCTTGGCTGTG-3’ |
| *Osr36* | AP001551 | 5’-CCCTGAATCCACCAAGAAAA-3’ | 5’-GGCAGTCTCGAGAAGGTGAC-3’ |
| *Osr42* | AF458768 | 5’-CCACAGATCATCATTTCTGACC-3’ | 5’-CCCCTTGAAGACTGACTTGC-3’ |
| *Tos17* | AC087545 | 5’-GCTACCCGTTCTTGGACTAT-3’ | 5’-CTGAAATCGGAGCACTGACA-3’ |
| *Tos19* | T03721 | 5’-TCTCAGCCTCCCACTTGTCT-3’ | 5’-TGTCCCATTGCCACATCTAA-3’ |
| *Lullaby* | AP008212 | 5’-CACAATTCACATGGTATCAGAG-3’ | 5’-GTGACTGTAGACGAGTTTCCT-3’ |
