# Supplementary material for: Immediate Genetic and Epigenetic Changes in F1 Hybrids Parented by Species with Divergent Genomes in the Rice Genus (Oryza)
Source: PLoS One. 2015 Jul 24;10(7):e0132911. doi: 10.1371/journal.pone.0132911 (PMC4514751; doi:10.1371/journal.pone.0132911)
Supplement: S5 Table — (DOC) [file pone.0132911.s005.doc]

**Table S5.** RT-PCR primers used in this study

| **Name** | **Genebank accession** | **Forward primer Sequence** | **Reverse primer Sequence** |
| --- | --- | --- | --- |
| *Ping* | AB087616 | 5’-GGCGTCCCACTGTAGAAGA-3' | 5’-AGAATCAGCGTTGGCACTT-3' |
| *Pong* | BK000586 | 5’-TTGGATGAGTTCCTGGCTGAG-3' | 5’-TGGACTGAGTCCTTTCCGATTA-3' |
| *Osr2* | AL442110 | 5’- AGATTTGGCAGAGGAGGTGTATG-3' | 5’-ACGAGTAGAGCAGAGCCACCA-3' |
| *Osr3* | AF458765 | 5’-CAAAGGCTGGTTGGGAGAC-3' | 5’-TCACTGACATTGCCCGATA-3' |
| *Osr7* | AP002538 | 5’-GCACCTCTGAGTGACCTTACCAT-3' | 5’-CACTCTTCTTCTTCAACTTGGGAC-3' |
| *Osr23* | AP002843 | 5’-GATGGCATAGTCGTCTTGGTCA-3' | 5’-TCAGATAAACAGCGGAAAGGAA-3' |
| *Osr35* | AC068924 | 5’-GCCATAGTAGCGGAAAGCCA-3' | 5’-CAGCAGCAAATGAAGCACCAG-3' |
| *Osr36* | AP001551 | 5’-ATCAGCCTTGTTCTTCATACGCAG-3' | 5’-TGGCATCCCTCGTTCTATTGTG-3' |
| *Osr42* | AF458768 | 5’-CCACAGATCATCATTTCTGACC-3’ | 5’-CCCCTTGAAGACTGACTTGC-3’ |
| *Tos17* | AC087545 | 5’-GCTACCCGTTCTTGGACTAT-3’ | 5’-CTGAAATCGGAGCACTGACA-3’ |
| *Lullaby* | AP008212 | 5’-CCTCCATCCTTTGCTGTCG-3' | 5’-AGGAAGCCCACTCACGGTAAT-3' |
| *OsActin* | X79378 | 5’-CGTGTGCGATAATGGAACTG-3' | 5’-TCTGGGTCATCTTCTCACGA-3' |
